# Recommendation of long-term and systemic management according to the risk factors in rectal NETs patients

**DOI:** 10.1038/s41598-018-37707-z

**Published:** 2019-02-20

**Authors:** Motohiro Kojima, Yu Chen, Koji Ikeda, Yuichiro Tsukada, Daigoro Takahashi, Shingo Kawano, Kota Amemiya, Masaaki Ito, Rieko Ohki, Atsushi Ochiai

**Affiliations:** 1 Division of Pathology, Exploratory Oncology Research & Clinical Trial Center, National Cancer Center, 6-5-1, Kashiwanoha, Kashiwa, Chiba 277-8577 Japan; 2 Laboratory of Fundamental Oncology, National Cancer Center Research Institute, Tsukiji 5-1-1, Chuo-ku, Tokyo 104-0045 Japan; 3 Division of Surgical Oncology, National Cancer Center Hospital East, 6-5-1, Kashiwanoha, Kashiwa, Chiba 277-8577 Japan; 4 Advanced Clinical Research of Cancer, Juntendo University Graduate School of Medicine, 3-1-3, Hongo, Bunkyo-ku, Tokyo 113-8431 Japan

## Abstract

Rectal neuroendocrine tumors (NETs) are often found as small lesions, which can be treated by endoscopic resection. However, high risk cases with lymph node (LN) metastasis are indication of radical surgery. Furthermore, rectal NETs are often associated with late recurrences and/or multiple cancer development. Therefore, proper surgical indication and patients’ management are required. We investigated the clinicopathological features of 79 rectal NET cases in order to elucidate risk factors for synchronous LN metastasis, recurrence, and multiple cancers. Recently, we reported that in pancreatic NET patients, a loss of heterozygosity (LOH) in *PHLDA3* was associated with poorer prognosis, and that LOH of both *PHLDA3* and *MEN1* was frequently observed. Therefore, *PHLDA3* and *MEN1* LOH were also assessed in rectal NET patients for their association with clinicopathological features. Of the 79 patients, LN metastases were found in 12.7%, recurrences in 3.8%, and multiple cancers in 30.4% of the subjects. *PHLDA3* and *MEN1* LOH were found in 60.0% and 66.7% of the subjects, respectively. Lymphatic invasion and WHO classification 2010 were found to be independent risks for LN metastasis. There were three cases of recurrence, all of which occurred more than 3 years after resection and two of which exhibited LN metastasis. Older age and LOH in *PHLDA3* were associated with the presence of multiple cancers. Long-term and systemic management of patients with rectal NETs is therefore recommended in accordance with these risk factors.

## Introduction

Due to the increase in colonoscopy screening, the incidence of rectal neuroendocrine tumors (NETs) has been rising, as is the incidence of other NETs throughout the body^1–3^. In many cases, rectal NETs are found as small lesions measuring less than 10 mm, and if they are not accompanied by lymph node metastasis, they are generally amenable to treatment by endoscopic resection. Resectable rectal NETs are associated with better prognosis than NETs in other organs. On the other hand, cases with possible lymph node metastasis may require radical surgery with lymph node dissection, which can result in anal dysfunction or permanent stoma. Therefore, accurately predicting lymph node metastasis by endoscopic and pathological examination is important. It is reported that tumor size, depth of invasion, presence of lymphovascular invasion, presence of central depression and Ki-67 index are associated with lymph node metastasis, and these factors can therefore influence the therapeutic strategy chosen^2–6^. On the other hand, some rectal NETs, including small lesions without vascular invasion, may have metastatic potential^3,7^. Therefore, further investigation is required to clarify risk factors for lymph node metastasis. Recently, we have reported that *PHLDA3* is a novel tumor suppressor and loss of *PHLDA3* heterozygosity is associated with poorer prognosis in pancreatic NET patients^8,9^. *MEN1* is another tumor suppressor gene frequently inactivated in pancreatic NET, and we previously observed that double loss of heterozygosity (LOH) of *PHLDA3* and *MEN1* frequently occurs in pancreatic NET^8,9^. Identifying such markers may contribute to decisions for rectal NET treatment^10^. Next, although resectable rectal NETs generally have a favorable clinical course, it is reported that they also are associated with an increased risk of secondary cancers^1,11^. Several cases of late recurrence have been reported^12,13^. Therefore, an accurate clinicopathological analysis of rectal NETs based on long-term follow-up data is needed to help establish a more standardized clinical approach to treat rectal NETs. In this study, we have examined the clinicopathological features of rectal NETs for their association with synchronous lymph node metastasis, recurrence, and the occurrence of multiple cancers. These features were also examined for their association with *PHLDA3* and *MEN1* LOH.

## Results

### Patient and tumor characteristics

**Figure 1:**
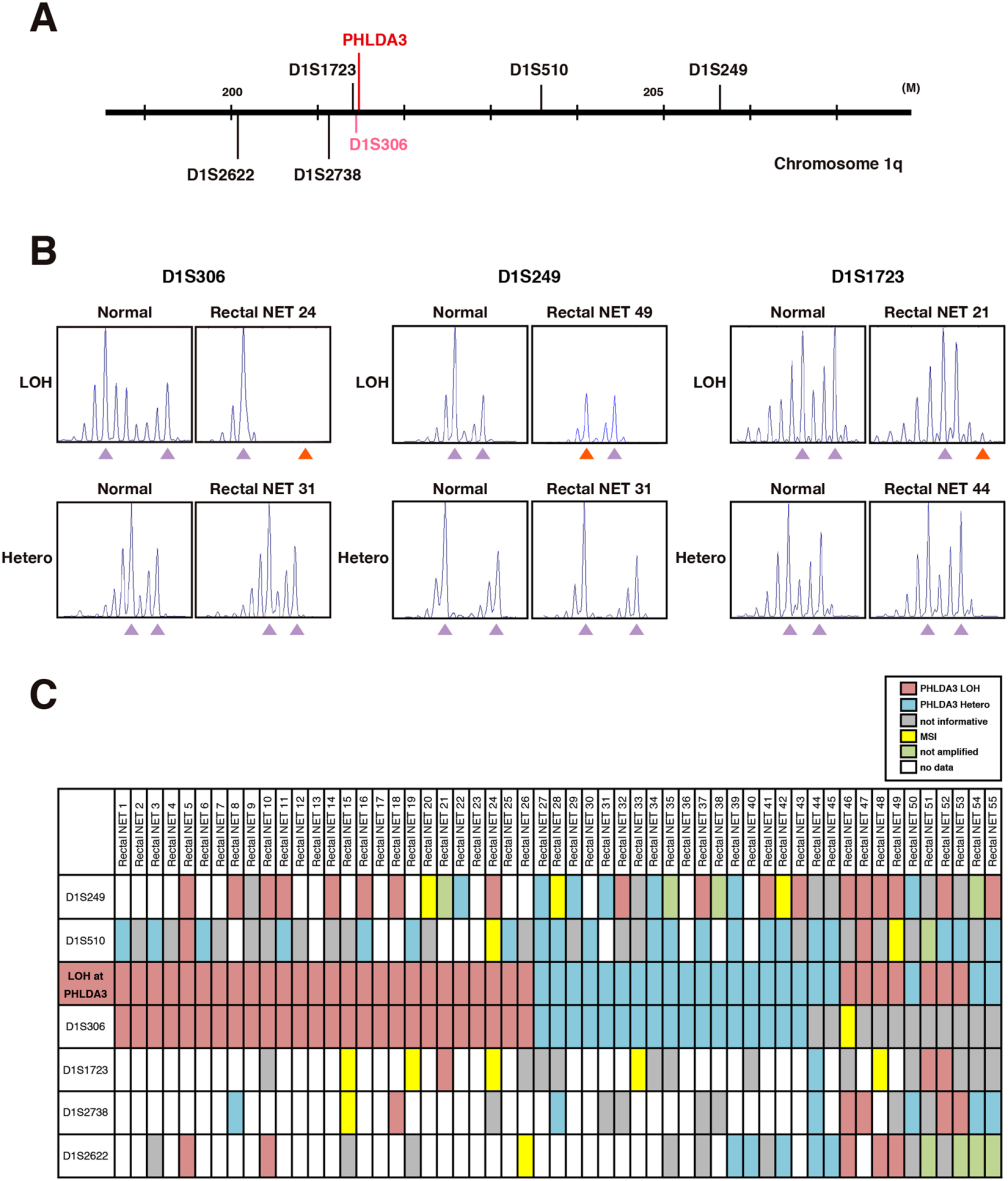
Frequency of LOH at the *PHLDA3* gene locus in rectal NETs. (**A**) Chromosomal locations of *PHLDA3* gene and microsatellite markers used in this study. D1S306 is located just next to the *PHLDA3* gene (32 kb upstream). (**B**) Representative microsatellite analysis results of *PHLDA3* gene. In normal tissues, two peaks derived from maternal and paternal alleles were detected, whereas in tumors, one allele was lost or changed (shown by orange arrows), indicating LOH at the locus. (**C**) Microsatellite analysis of the *PHLDA3* gene locus region. Rectal NET samples were analyzed for LOH around the *PHLDA3* gene locus. Because D1S306, D1S1723 and D1S2738 are located near to the *PHLDA3* gene, the LOH status of the *PHLDA3* gene was determined from the LOH status of these markers. For samples with no data for these markers, the LOH status of the locus was determined from the surrounding LOH status. In some cases, LOH status was unavailable because of failure of PCR due to insufficiency or low quality of DNA (shown in green columns), microsatellite instability (shown in yellow columns) or homozygosity (shown in gray columns).

**Figure 2:**
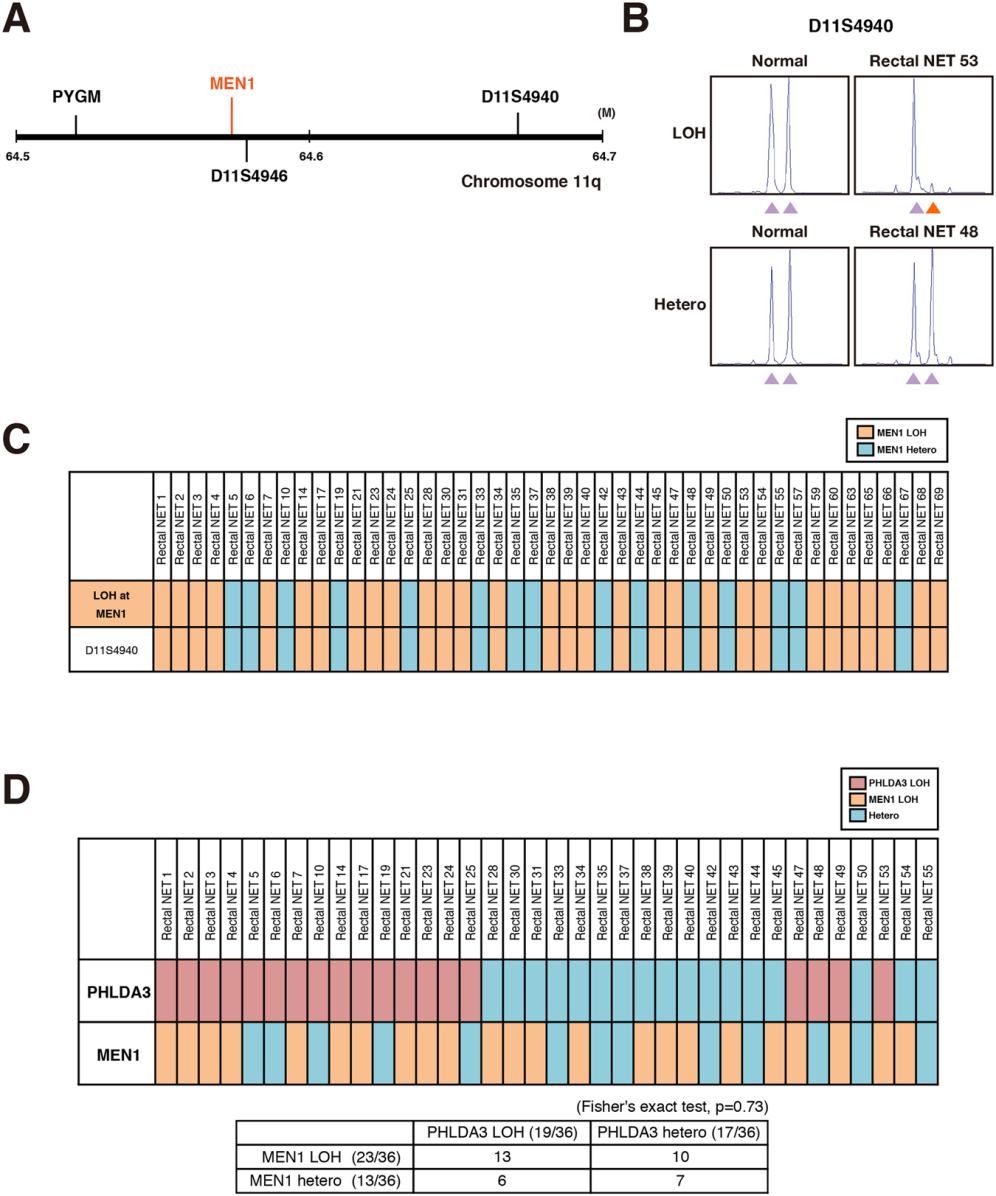
Frequency of LOH at the *MEN1* gene locus in rectal NETs. (**A**) Chromosomal locations of the *MEN1* gene and microsatellite marker used in this study. (**B**) Representative microsatellite analysis results of *MEN1* gene. (**C**) Microsatellite analysis of LOH at the *MEN1* gene locus. The LOH status of the *MEN1* gene was determined from the LOH status of D11S4940, D11S4946 or PYGM. (**D**) Relationship between LOH status of the PHLDA3 and MEN1 loci. In total, 36 samples informative for both PHLDA3 and MEN1 loci were analyzed.

The clinicopathological features of 79 patients with rectal NET are shown in Table 1. Rectal NETs were found to occur predominantly in males (73.4%; n = 58); the mean age of the patients was 58.5 ± 12.8 years; and all patients were Asian. Of the 20 patients undergoing surgery, lymph node metastases were found in 10 and assessed as N1 (12.7%), and the tumor size averaged 9.6 ± 9.1 mm (consisting of <10 mm size in 51 patients [64.6%], 10–20 mm in 21 patients [26.6%], and >20 mm in 7 patients [8.9%]). All cases were NET G1 and G2 according to WHO classification 2010, and NET G3 were not included in this study. Lymphatic invasion was found in 13 cases (16.5%) and venous invasion in 22 cases (27.8%). We also analyzed LOH of *PHLDA3* and *MEN1*, two tumor suppressor genes frequently inactivated in pancreatic NETs. LOH analysis of *PHLDA3* was successfully performed in 55 cases, of which 60.0% (33/55) were positive, and LOH analyses of *MEN1* was successful in 45 cases, of which 66.7% (30/45) were positive (Figs 1 and 2). The strikingly high incidence of LOH at these loci indicates that *PHLDA3* and *MEN1* are important in tumor suppression in rectal NETs. We next combined the LOH data for both loci. As shown in Fig. 2D, LOH at the *PHLDA3* and *MEN1* loci did not show a mutually exclusive pattern, which would be expected if *PHLDA3* and *MEN1* were on the same tumor suppressing pathway. Interestingly, a high frequency of double LOH, i.e. occurring at both the *PHLDA3* and *MEN1* loci was observed, although the frequency did not reach statistical significance (13 out of 36 samples were double-positive, p = 0.73 by Fisher’s exact test). These data suggest that the *PHLDA3* and *MEN1* tumor suppressing pathways are distinct, and that rectal NET development involves the functional loss of both pathways. As shown in Fig. S1, determination of LOH status was unsuccessful in 24 cases for *PHLDA3* and 34 cases for *MEN1*. This was due to a failure in PCR amplification caused by low quality or insufficient DNA amounts (green columns), microsatellite instability (yellow columns), homozygosity (gray columns), or DNA sample was unavailable (pink columns).

**Table 1:** Clinicopathological features of rectal NETs.

Gender	Male (%)	58 (73.4%)
	Female (%)	21 (26.6%)
Age (years)	Mean ± SD	58.5 ± 12.8
AJCC stage	Stage I	66 (83.5%)
	Stage II	3 (3.8%)
	Stage III	9 (11.4%)
	Stage IV	1 (1.3%)
T stage	T1	73 (92.4%)
	T2,3	6 (7.6%)
N stage	N0	69 (87.3%)
	N1	10 (12.7%)
Tumor size (mm)	Mean ± SD	9.6 ± 9.1
	<10 mm	51 (64.6%)
	10–20 mm	21 (26.6%)
	≥20 mm	7 (8.9%)
WHO classification 2010	NET G1	72 (91.1%)
	NET G2	7 (8.9%)
Mitotic count (/10HPF)	<2	74 (93.7%)
	2–20	5 (6.3%)
Ki-67 index (%)	≤2	77 (97.5%)
	3–20	2 (2.5%)
Lymphatic invasion	Positive	13 (16.5%)
	Negative	66 (83.5%)
Venous invasion	Positive	22 (27.8%)
	Negative	57 (72.2%)
PHLDA3 LOH	Positive	33 (60.0%)
	Negative	22 (40.0%)
MEN1 LOH	Positive	30 (66.7%)
	Negative	15 (33.3%)
Recurrence	Positive	3 (3.8%)
	Negative	76 (96.2%)
Disease-specific death	Positive	2 (2.5%)
	Negative	77 (97.5%)
Multiple cancers	Positive	24 (30.4%)
	Negative	55 (69.6%)

Abbreviations: NET, neuroendocrine tumor; HPF, high power field.

### Clinicopathological features associated with lymph node metastasis, recurrence, and multiple cancers

Synchronous lymph node metastases were found in 10/79 cases (12.7%). The associations between various clinicopathological features and lymph node metastasis are shown in Table 2. Both WHO classification 2010 and lymphatic vessel invasion were found to be independent risk factors for lymph node metastasis. *MEN1* and *PHLDA3* LOH were not associated with lymph node metastasis. The clinicopathological features of patients with lymph node metastasis are shown in Supplementary Table S1. All cases had at least one of following risk factors; tumor size >10 mm, lymphatic invasion, venous invasion, or G2 in the WHO classification 2010.

**Table 2:** Comparison of characteristics between cases with and without lymph node metastasis.

	With lymph node metastasis (n = 10) (%)	Without lymph node metastasis (n = 69) (%)	Univariate	Multivariate
			*P* value	*P* value
**Gender**				
Male	7 (70.0%)	51 (73.9%)	NS	
Female	3 (30.0%)	18 (26.1%)		
**Age (years)**	57.9 ± 11.1	58.6 ± 13.1	NS	
**WHO classification 2010**				
NET G1	5 (50.0%)	67 (97.1%)	0.01>	0.01>
NET G2	5 (50.0%)	2 (2.9%)		
**Mitotic count (/10HPF)**				
<2	6 (60.0%)	68 (98.6%)	0.01>	
2–20	4 (40.0%)	1 (1.4%)		
**Ki-67 index (%)**				
≤2	9 (90.0%)	68 (98.6%)	0.239	
3–20	1 (10.0%)	1 (1.4%)		
**Tumor size**				
Mean, mm	22.3 ± 17.6	7.8 ± 5.3	0.029	0.06
**Depth of tumor invasion**				
submucosa	7 (70.0%)	68 (98.6%)	0.01>	
Muscular layer or deeper	3 (30.0%)	1 (1.4%)		
**Lymphatic invasion**				
Positive	7 (70.0%)	6 (8.7%)	0.01>	0.01>
Negative	3 (30.0%)	63 (91.3%)		
**Venous invasion**				
Positive	8 (80.0%)	14 (20.3%)	0.01>	0.21
Negative	2 (20.0%)	55 (79.7%)		
**MEN1 LOH (n** = **45)**				
Positive	6 (75.0%)	24 (64.9%)	NS	
Negative	2 (25.0%)	13 (35.1%)		
**PHLDA3 LOH (n** = **55)**				
Positive	3 (33,3%)	30 (65.2%)	NS	
Negative	6 (66.7%)	16 (34.8%)		
**Recurrence**				
Positive	2 (20.0%)	1 (1.4%)	0.041	
Negative	8 (80.0%)	68 (98.6%)		
**Multiple cancers**				
Positive	2 (20.0%)	22 (31.9%)	NS	
Negative	8 (80.0%)	47 (68.1%)		

Abbreviations: NET, neuroendocrine tumor; HPF, high power field; LOH, loss of heterozygosity; NS, not significant.

The mean follow-up of the patients included in the analysis was 2136.0 ± 1472.9 days. During follow-up, recurrences occurred in 3 patients (3.1%), and 2 of these dying of the disease (Table 3). All cases were classified as G2 in the WHO classification 2010 and were shown to be positive for *PHLDA3* LOH. Synchronous lymph node metastasis was found in 2 of the 3 recurrent cases. Of the 3 recurrent cases, 2 had liver metastases and 1 had a local recurrence. The time to recurrence from resection in these 3 recurrent cases was 1798, 1916, and 2595 days, respectively. And, all recurrences occurred over a 3-year period following NET resection.

**Table 3:** Cases with recurrences and disease-specific death.

Age	Gender	Resection	pT (tumor size)	pN	Lymphatic invasion	Venous invasion	WHO classification 2010	MEN1 LOH	PHLDA3 LOH	Site of recurrence	Follow-up before recurrence	Total follow-up	Clinical outcome
61	Male	Radical	3 (55 mm)	1	(+)	(+)	Grade 2	(+)	(+)	Liver	1916 days	5550 days	Died of disease
66	Female	Radical	3 (55 mm)	1	(+)	(+)	Grade 2	NA	(+)	Liver	2595 days	3162 days	Lost to follow-up
70	Male	Local	1 (10 mm)	0	(−)	(−)	Grade 2	(+)	(+)	Local	1798 days	3781 days	Died of disease

Abbreviations: NA, data not available.

Table 4 and Supplementary Table S2 list the clinicopathological features of rectal NETs presenting with multiple cancers. Multiple cancers were detected in 24 of the 79 cases (30.4%). Of these, 5 cases had multiple lesions involving 2 organs (2 cases involving the stomach and colorectum, 2 involving the colorectum and lung, and 1 case involving the esophagus and stomach). Of the 29 tumors, 23 were histologically confirmed, 5 were recorded as a past history, and 1 case of pancreatic cancer was detected radiographically. Multiple colorectal cancers were found in 10 subjects, gastric cancer in 4, esophageal cancer in 3 (all squamous cell carcinoma), lung cancer in 3, prostate cancer in 2, and liver cancer in 2 subjects (1 hepatocellular carcinoma and 1 cholangiocellular carcinoma). One case involved lesions of the skin (malignant melanoma), kidney, pancreas, and breast. In addition, one gastric GIST was found. Fifteen tumors were found concurrent with rectal NETs, with 10 multiple tumors detected over 6 months before the resection of the rectal NETs. Four multiple tumors were detected over 6 months after removal of the rectal NET. The occurrence of multiple cancers was significantly associated with older age and the presence of *PHLDA3* LOH, but not with any other clinicopathological feature. Importantly, 5 patients died of multiple cancers other than rectal NETs during the follow-up period.

**Table 4:** Comparison of characteristics of patients with and without multiple cancers.

	Patients with multiple cancer	Patients without multiple cancer	*P* value
**Gender**			
Male	18 (75.0%)	40 (72.7%)	NS
Female	6 (25.0%)	15 (27.3%)	
**Age (years)**	64.5 ± 8.7	55.9 ± 13.5	0.01>
60 years old ≤	18 (75.0%)	24 (43.6%)	0.01
60 years old>	6 (25.0%)	31 (56.4%)	
**pT stage**			
pT1	21 (87.5%)	52 (94.5%)	NS
pT23	3 (12.5%)	3 (5.5%)	
**pN stage**			
pN0	22 (91.7%)	47 (85.5%)	NS
pN1	2 (8.3%)	8 (14.5%)	
**Tumor size**			
Mean (mm)	10.3 ± 11.4	9.3 ± 8.1	NS
**WHO classification 2010**			
NET G1	22 (91.7%)	50 (90.9%)	NS
NET G2	2 (8.3%)	5 (9.1%)	
**Lymphatic invasion**			
Positive	4 (16.7%)	9 (16.4%)	NS
Negative	20 (83.3%)	46 (83.6%)	
**Venous invasion**			
Positive	6 (25.0%)	16 (29.1%)	NS
Negative	18 (75.0%)	39 (70.9%)	
**MEN1 LOH (n** = **45)**			
Positive	7 (63.6%)	23 (67.6%)	NS
Negative	4 (36.4%)	11 (32.4%)	
**PHLDA3 LOH (n** = **55)**			
Positive	14 (82.4%)	19 (50.0%)	0.02
Negative	3 (17.6%)	19 (50.0%)	
**Recurrence**			
Positive	1 (4.2%)	2 (3.6%)	NS
Negative	23 (95.8%)	53 (96.4%)	

Abbreviations: NET, neuroendocrine tumor; LOH, loss of heterozygosity; NS, not significant.

The association between LOH and various clinicopathological features is shown in Table 5. The observed frequency of *PHLDA3* LOH in rectal NET (60.0%) was similar to the reported frequency in pancreatic NET (72.0%)^8^. Of the various clinicopathological features recorded, *PHLDA3* LOH was associated only with the occurrence of multiple cancers. *MEN1* LOH was found in 66.7% of the rectal NET, and was not associated with any clinicopathological features.

**Table 5:** Association between PHLDA3, MEN1 LOH and clinicopathological features.

	PHLDA3 LOH (+) (%)	PHLDA3 LOH (−) (%)	*P* value	MEN1 LOH (+) (%)	MEN1 LOH (−) (%)	*P* value
**Gender**						
Male	26 (78.8%)	16 (72.7%)	NS	24 (80.0%)	9 (60.0%)	0.15
Female	7 (21.2%)	6 (27.3%)		6 (20.0%)	6 (40.0%)	
**Mean age**, **years**	60.0 ± 12.2	56.1 ± 14.1	NS	58.3 ± 10.1	51.3 ± 15.8	NS
**pT Stage**						
pT1	29 (87.9%)	22 (100%)	NS	28 (93.3%)	13 (86.7%)	NS
pT23	4 (12.1%)	0 (0%)		2 (6.7%)	2 (13.3%)	
**pN Stage**						
pN0	30 (90.9%)	16 (72.7%)	NS	24 (80.0%)	13 (86.7%)	NS
pN1	3 (9.1%)	6 (27.3%)		6 (20.0%)	2 (13.3%)	
**WHO classification 2010**						
NET G1	30 (90.9%)	20 (90.9%)	NS	26 (86.7%)	14 (93.3%)	NS
NET G2	3 (9.1%)	2 (9.1%)		4 (13.3%)	1 (6.7%)	
**Tumor size**						
Mean (mm)	9.5 ± 9.9	9.1 ± 4.7	NS	9.4 ± 9.7	10.1 ± 7.0	NS
**Recurrence**						
Positive	2 (6.1%)	0 (0%)	NS	1 (3.3%)	0 (0%)	NS
Negative	31 (93.9%)	22 (100%)		29 (96.7%)	15 (100%)	
**Multiple cancers**						
Positive	14 (42.4%)	3 (13.6%)	0.02	7 (23.3%)	4 (26.7%)	NS
Negative	19 (57.6%)	19 (86.4%)		23 (76.7%)	11 (73.3%)	
**Lymphatic invasion**						
Positive	5 (15.2%)	5 (22.7%)	NS	6 (20.0%)	2 (13.3%)	NS
Negative	28 (84.8%)	17 (77.3%)		24 (80.0%)	13 (86.7%)	
**Venous invasion**						
Positive	9 (27.3%)	7 (31.8%)	NS	10 (33.3%)	5 (33.3%)	NS
Negative	24 (72.7%)	15 (68.2%)		20 (66.7%)	10 (66.7%)	

Abbreviations: NET, neuroendocrine tumor; HPF, high power field; LOH, loss of heterozygosity; NS, not significant.

## Discussion

Consistent with earlier reports, resectable rectal NETs showed a favorable clinical course in our study, with a recurrence rate of only 3.1% and a disease-specific survival of 96.9% during the mean follow-up period over 5 years. There were no recurrences in the first three years after resection, one after three years and two after five years. Of the 3 recurring cases, 2 had synchronous lymph node metastases. Therefore, long-term follow up may be especially required in patients showing synchronous lymph node metastasis. In addition, 30.4% of the patients were found to have multiple cancers other than NET, the likelihood of which was associated with older age and the presence of *PHLDA3* LOH. Previous epidemiological studies have also reported an association with multiple tumors^1,11^. In a long-term follow-up at our institution, we also confirmed a high incidence of multiple tumors in various organs among these patients. To our knowledge, the data presented here is the first to describe the clinicopathological features of patients suffering from rectal NETs associated with multiple cancers. Many of the cases of multiple cancers were found prior to or at the same time as the diagnosis of rectal NET. In particular, our results suggest that comprehensive screening for multiple cancers is especially recommended for patients over 60 years of age. Biological markers that would predict the recurrence of NETs or the presence of multiple cancers would contribute to more efficient patient management, and *PHLDA3* LOH may constitute one such marker to predict a higher probability of multiple cancers^10^. In this regard, higher gene methylation levels in normal tissue have been reported to be associated with metachronous gastric cancer^14^. While the role of *PHLDA3* in carcinogenesis is less clear, higher level of epigenetic alterations in normal tissues may also be found in rectal NETs patients. It is likely that LOH of *PHLDA3* in rectal NETs results in the production of some tumor promoting factors, but further study will be required to confirm this. In addition, we note that the most of multiple tumors we observed were in the colon, rectum, stomach, and these organs are differentiated from endoderm. We therefore speculate that the *PHLDA3* LOH must have occurred in endoderm cell at a very early stage of the development. It is well-known that Li-Fraumeni syndrome patients, who have germline p53 mutation, also develop multiple cancers. *PHLDA3* is a target gene and important down-stream mediator of p53, therefore dysfunction of PHLDA3 at an early stage of development may also contribute to multiple cancer formation.

It is nortworthy that in pancreatic endocrine tissue, *PHLDA3* acts as a tumor suppressor, and methylation and/or LOH of *PHLDA3* activates Akt-regulated biological processes. *PHLDA3* status has also been reported to be associated with clinical outcome in pancreatic NET patients. Although we observed no association between *PHLDA3* LOH and lymph node metastasis, all three cases of recurring NETs were found to have *PHLDA3* LOH. Thus, further study is required to evaluate the clinical utility of *PHLDA3* LOH in predicting tumor behavior. In addition, rectal NETs are not usually associated with the *MEN1* syndrome, and *MEN1* LOH has been thought to be rare^15^. However, previous reports analyzed only 1 or 2 patients, so reliable epidemiological data was not available^16–18^. Therefore, we investigated LOH of *MEN1* as well as *PHLDA3* to estimate the clinical utility of each as a biomarker. We report here the novel finding that LOH occurs at the *PHLDA3* and *MEN1* loci in 60.0% and 66.7% of rectal NETs cases, respectively. Using the LOH analysis method established in previous studies, we identified a slightly higher frequency of *MEN1* LOH compared to *PHLDA3* LOH in rectal NETs^8^. Thus, this is the first report to assess the frequency of LOH at the *MEN1* locus and show its association with *PHLDA3* LOH. Consistent with our previous report on pancreatic NETs, patients with rectal NETs frequently exhibited double LOH, which differed from matually exclusive pattern between *K-RAS* and *BRAF* mutations in colorectal cancer^6^. Further study will be required to confirm generality of our results from single institution. In addition, although most rectal NETs are thought to be sporadic without genetic disorder, some rectal NET with neurofibromatosis or Peutz-Jeghers syndrome has also been reported. Therefore, the molecular biological analysis of genes related to these diseases may also provide useful findings^19–21^.

One case showed local recurrence after trans-anal excision, thus it is important to clearly understand the parameters that might indicate radical surgery in the management of rectal NETs. Tumor size is a simple and objective measure to guide therapeutic decision proposed by the ENETS and NCCN guidelines^22,23^. Although there are some reported exceptions, lesions measuring < 10 mm in diameter, and without lympho-vascular invasion have been reported to generally have a low risk of metastatic disease and therefore can be resected endoscopically or by other local transanal resections^3^. Tumors > 20 mm, T3 or T4 stage, and having G3 grading by the ENETS guidelines or those with loco-regional lymph node involvement should indicate the need for additional surgical resection of the rectum with mesorectal lymph node dissection. On the other hand, for tumors measuring between 10 and 20 mm, the recommendations provided by the ENETS and NCCN guidelines are ambiguous, and their metastatic risk is considered to be between 10% and 15%. Our retrospective study showed that 8 of the 10 patients with lymph node metastasis had tumors measuring ≤ 20 mm in size. Five of these patients with lymph node metastasis fulfilled the criteria, including, tumor size ≤ 20 mm, NET G1 and submucosal invasion in these guidelines. These 5 cases could be observed after endoscopic resection or trans anal excision. However, 4 of these cases were shown to have had lymphatic or venous invasion in pathological examinations, which lead us undertake radical surgery. Therefore, a combined assessment of histological factors, WHO grading, lymphatic vessel invasion, and venous invasion should be used to guide the decision to undertake additional surgical resection in tumors sizes between 10 and 20 mm. Although vascular invasion is reported to be a less objective measure, the concordance of vascular invasion in pT1 colorectal cancers has reported to be 0.52–0.56^24,25^. Therefore, an assessment of vascular invasion in rectal NETs, most of which are small and within submucosa, may provide increased objectivity in determining a course of treatment. However, further pathological study is required to confirm this point.

In conclusion, we believe that special attention should be given to recurrences that are found more than 3 years after resection, especially in case showing synchronous lymph node metastasis. The risk for multiple cancers should be also considered especially in patients over 60 years of age and those exhibiting *PHLDA3* LOH. Lymphatic invasion and the WHO classification 2010 were found to be good predictors of synchronous lymph node metastasis, and the combined use of these factors may assist in determining surgery after local resection. Rectal NET patients appear to require systemic screening before resection, as well as long-term and systemic management after resection. Biological and biomarker research focused on recurrence or multiple cancers would be beneficial for the long term management of rectal NET patients.

## Materials and Methods

### Informed consent

All experiments were performed after obtaining written comprehensive informed consents from all patients. This study was approved by the National Cancer Ethical Review Board (No. 2013-032), and was performed in accordance with relevant guidelines and regulations.

### Patients

Using pathological data base, we enrolled 79 patients retrospectively diagnosed as rectal NET G1 and G2 according to the World Health Organization (WHO) classification 2010 between January 1, 1999 and March 31, 2014 at National Cancer Center East Hospital^15^. All cases were intended for curative resection. Endoscopic resections, transanal excision, and radical surgery were underwent in 54 (68.4%) cases, 5 (6.3%) cases, and 20 (25.3%) cases, respectively. Additional surgical resection of the rectum with mesorectal lymph node was considered after endoscopic resection if pathological risk factor such as a positive margin, tumor size ≥10 mm, invasion into the muscularis propria, the presence of lympatic and venous invasion or NETG2 were identified. Of the 20 patients undergoing radical resection, 5 underwent additional resection after initial endoscopic resection. All patients underwent colonoscopy and an abdominopelvic computed tomography (CT) scan to evaluate the presence of metastatic disease before resection. Regarding the postoperative course of the patient, we conducted an outpatient visit once every four months for the first two years, and once every six months for the following three years. At the time of follow-up, we conducted an examination (chest, abdomen, pelvic CT examination and blood sampling test) according to the protocol of the Japan Colon Cancer Research Group. In addition, we performed a lower gastrointestinal endoscopy in the 2nd and 5th postoperative periods. After 5 years postoperative surgery, patients’ visit was taken once a year. Clinical information were reviewed and recorded retrospectively including age, sex, method of resection used, recurrence, synchronous and metachronous multiple cancers other than neuroendocrine tumors. Multiple cancers detected 6 months before and after the diagnosis of rectal NETs were defined as synchronous lesions, with all other lesions defined as metachronous lesions^11^. Cancer staging was performed using the 7^th^ edition of the American Joint Committee on Cancer (AJCC) Staging Manual^26^.

### Histological diagnosis

All specimens were pathologically reviewed and assessed retrospectively by two investigators (K.I and M.K). Maximum tumor size was determined based on H.E. slides and neuroendocrine differentiation was assessed by positive immunohistochemical staining for chromogranin A (diluted 1:200, clone DAK-A3, Dako, Glostrup, Denmark), synaptophysin (ready to use, DAK-A3, Dako, Glostrup, Denmark), and CD56 in all cases (diluted 1:50, clone 123C3, Dako, Glostrup, Denmark). Tumor specimens were considered positive for neuroendocrine markers if more than 5% of tumor cells were stained. All tumors are positive for at least two of three neuroendocrine markers. Elastica and D2–40 staining was used in all cases to assess lymph-vascular invasion (diluted 1:200, clone D2–40, Acris, Herford, Germany). The Ki-67 and mitotic index was evaluated, and grading was performed according to the WHO classification 2010^15^. Lymphatic invasion and venous invasion were assessed as reported previously^27^. Lymph node metastasis was confirmed histologically using surgical specimens, and distant metastases were evaluated either radiologically or histologically.

### LOH analysis

LOH analysis of *MEN1* and *PHLDA3* loci were performed as reported previously^8^. Five 10 μm-thick slides from formalin-fixed paraffin-embedded tumors were used for LOH analysis. Six primer pairs labeled with FAM with amplified microsatellite loci were used to accurately detect LOH at the *PHLDA3* locus. For the *MEN1* locus, 3 primer pairs were used. Amplified PCR products were analyzed with a 3100 automated sequencer and GeneScan and Genotyper software (Applied Biosystems, Foster City, CA, USA). The genotype was determined to be heterozygous if two bands of different sizes were obtained from normal tissues. A ratio of the two peaks in tumor DNA of less than 0.7 in comparison with the corresponding ratio of the two peaks in non-tumor DNA was considered as allelic loss. Primers used in this study are shown in Supplementary Table S3.

### Statistical analysis

Continuous variables, such as age and tumor size, were expressed as mean ± SD. Their association with synchronous lymph node metastases, multiple cancer, *MEN1* LOH, and *PHLDA3* LOH were assessed using Student’s *t*-test. Other non-continuous clinicopathological characteristics were assessed with χ^2^ and Fisher’s exact tests. Multiple logistic regression analysis was performed on independent risk factors for synchronous lymph node metastasis. All analyses were performed using statistical software Minitab (Kozo Keikaku Engineering Inc., Tokyo, Japan).

## Supplementary information

Supplementary Information
