# Supplementary material for: Recommendation of long-term and systemic management according to the risk factors in rectal NETs patients
Source: Sci Rep. 2019 Feb 20;9:2404. doi: 10.1038/s41598-018-37707-z (PMC6382938; doi:10.1038/s41598-018-37707-z)
Supplement: Supplementary file 1 — Supplementary Information [file 41598_2018_37707_MOESM1_ESM.docx]

**Recommendation of long-term and systemic management according to the risk factors in rectal NETs patients.**

Motohiro Kojima^1^, Yu Chen^2^, Koji Ikeda^3^, Yuichiro Tsukada^3^, Daigoro Takahashi^3^, Shingo Kawano^4^, Kota Amemiya^4^, Masaaki Ito^3^, Rieko Ohki^2^, Atsushi Ochiai^1*^

^1^Division of Pathology, Exploratory Oncology Research & Clinical Trial Center, National Cancer Center, 6-5-1, Kashiwanoha, Kashiwa, Chiba, 277-8577, Japan

^2^Laboratory of Fundamental Oncology, National Cancer Center Research Institute, Tsukiji 5-1-1, Chuo-ku, Tokyo 104-0045, Japan

^3^Division of Surgical Oncology, National Cancer Center Hospital East, 6-5-1, Kashiwanoha, Kashiwa, Chiba, 277-8577, Japan

^4^Advanced Clinical Research of Cancer, Juntendo University Graduate School of Medicine, 3-1-3, Hongo, Bunkyo-ku, Tokyo, 113-8431, Japan

***Corresponding author:** Atsushi Ochiai **(**[aochiai@east.ncc.go.jp](mailto:aochiai@east.ncc.go.jp))

| **Supplementary Table S1 Characteristics of patients with lymph node metastasis** | | | | | | | | |
| --- | --- | --- | --- | --- | --- | --- | --- | --- |
| Age | Gender | pT (tumor size) | Lymphatic invasion | Venous invasion | WHO classification 2010 | MEN1 LOH | PHLDA3 LOH | Recurrence |
| 54 | Male | 1b (14mm) | Negative | Negative | Grade 1 | Negative | Negative | Negative |
| 35 | Female | 1b (13mm) | Negative | Positive | Grade 2 | Negative | Negative | Negative |
| 74 | Male | 1b (20mm) | Positive | Positive | Grade 1 | Positive | Negative | Negative |
| 52 | Male | 1b (10mm) | Positive | Negative | Grade 1 | Positive | Negative | Negative |
| 61 | Male | 3 (20mm) | Positive | Positive | Grade 2 | Positive | Positive | positive |
| 58 | Male | 1a (8mm) | Positive | Positive | Grade 1 | Positive | Positive | Negative |
| 60 | Male | 3 (55mm) | Positive | Positive | Grade 2 | Positive | Not available | Negative |
| 69 | Male | 1b (13mm) | Positive | Positive | Grade 1 | Not available | Negative | Negative |
| 66 | Female | 3 (55mm) | Positive | Positive | Grade 2 | Not available | Positive | Positive |
| 50 | Female | 1b (15mm) | Negative | Positive | Grade 2 | positive | Negative | Negative |

| Age | Gender | pT  (tumor size) | AJCC stage | WHO classification 2010 | MEN1 LOH | PHLDA3 LOH | Multiple cancer site 1 | Onset | Multiple cancer site 2 | Onset | Clinical outcome |
| --- | --- | --- | --- | --- | --- | --- | --- | --- | --- | --- | --- |
| 41 | Female | 1a (4 mm) | I | NET G1 | NA | NA | Colon | Synchronous |  |  | Alive |
| 47 | Male | 1a (5 mm) | I | NET G1 | NA | Positive | Prostate | 16 years later |  |  | Alive |
| 56 | Male | 1a (4 mm) | I | NET G1 | Positive | Positive | Rectum | Synchronous | Lung | 7 months later | Died of lung cancer |
| 57 | Female | 1a (4 mm) | I | NET G1 | Positive | Positive | Breast | 2 years earlier |  |  | Died of breast cancer |
| 58 | Male | 1a (8 mm) | I | NET G1 | NA | NA | Colon | 5 years earlier |  |  | Alive |
| 59 | Male | 1b (15 mm) | I | NET G1 | Negative | Positive | Stomach | Synchronous | Esophagus | 1 year later | Alive |
| 60 | Male | 3 (55 mm) | IV | NET G2 | Positive | NA | Liver | 4 years earlier |  |  | Died of liver cancer |
| 61 | Male | 3 (20 mm) | IIIB | NET G2 | Positive | Positive | Stomach | Synchronous |  |  | Died of NET |
| 64 | Male | 1a (6 mm) | I | NET G1 | Positive | Negative | Stomach | Synchronous | Colon | Synchronous | Died of cerebral infarction |
| 64 | Female | 1b (15 mm) | I | NET G1 | NA | Negative | Colon | Synchronous | Lung | 1 year later | Died of lung cancer |
| 64 | Male | 1a (4 mm) | I | NET G1 | NA | Positive | Esophagus | 2 years earlier | Stomach | 2 years later | Alive |
| 65 | Male | 1a (3 mm) | I | NET G1 | Negative | NA | Pancreas | Synchronous |  |  | Died of pancreas cancer |
| 66 | Female | 1b (10 mm) | I | NET G1 | NA | NA | Esophagus | 6 years earlier |  |  | Alive |
| 67 | Male | 1a (9 mm) | I | NET G1 | NA | Positive | Liver | Synchronous |  |  | Alive |
| 68 | Male | 1a (5 mm) | I | NET G1 | Positive | Positive | Esophagus | 10 months earlier |  |  | Alive |
| 68 | Male | 1a (5 mm) | I | NET G1 | NA | Positive | Colon | Synchronous |  |  | Alive |
| 69 | Female | 1a (4 mm) | I | NET G1 | Positive | Negative | Rectum | Synchronous |  |  | Alive |
| 69 | Male | 1a(9mm) | I | NET G1 | Negative | Positive | Stomach | 3years ealier |  |  | Alive |
| 72 | Male | 1b (10 mm) | I | NET G1 | NA | Positive | Stomach (GIST) | 2 years earlier |  |  | Alive |
| 72 | Male | 2 (30 mm) | II | NET G1 | NA | NA | Prostate | Synchronous |  |  | Alive |
| 74 | Male | 1a (7 mm) | I | NET G1 | NA | Positive | Colon | 5 years earlier |  |  | Alive |
| 75 | Male | 1a (4 mm) | I | NET G1 | NA | NA | Rectum | 2 years earlier |  |  | Alive |
| 76 | Female | 1a (4 mm) | I | NET G1 | Negative | Positive | Skin (melanoma) | Synchronous |  |  | Alive |
| 76 | Male | 1a (8 mm) | I | NET G1 | NA | Positive | Kidney | Synchronous |  |  | Alive |
| Abbreviations: NA, data not available; NET, neuroendocrine tumor | | | | | |  |  |  |  |  |  |

**Supplementary Table S2 Characteristics of patients with multiple cancers**

| **Suplementary Table S3** **Primers (6-FAM-labeled Primers)** | | | | |
| --- | --- | --- | --- | --- |
| **PHLDA3** | | | | |
| D1S306-Fwd-Primer Sequence: 5’6-FAM-CGATCTCAGGCATATAGTCAGTC | | | | |
| D1S306-Rev-Primer Sequence: 5’CCAGAGGGAGCATTGGTG | | | | |
|  | | | | |
| D1S249-Fwd-Primer Sequence: 5’6-FAM-TGGCATGTCTTTGAAGGAAT | | | | |
| D1S249-Rev-Primer Sequence: 5’TGGTTGTAGATGAGACTGGC | | | | |
|  | | | | |
| D1S510-Fwd-Primer Sequence: 5’6-FAM-TTCCTGCTCCTGTCTGAATA | | | | |
| D1S510-Rev-Primer Sequence: 5’TGTATATAAGGTGTAGGGGAGG | | | | |
|  | | | | |
| D1S2622-Fwd-Primer Sequence: 5’6-FAM-CTGCAACATAAGAACCTAGTGTAAC | | | | |
| D1S2622-Rev-Primer Sequence: 5’AAACTGGTAGGCCATTGATAGA | | | | |
|  | | | | |
| D1S1723-Fwd-Primer Sequence: 5’6-FAM-AACTGTGTCCAGCAGCAACT | | | | |
| D1S1723-Rev-Primer Sequence: 5’TATGTGCCTGTTGTGTGCAT | | | | |
|  | | | | |
| D1S2738-Fwd-Primer Sequence: 5’AACACATGCATACATACGACA | | | | |
| D1S2738-Rev-Primer Sequence: 5’6-FAM-GTCACATTTCAGGGCAGG | | | | |
|  | | | | |
|  | | | | |
| **MEN 1** |  |  |  |  |
| D1S4940-Fwd-Primer Sequence: 5’6-FAM-ACGTAGCAGTCCTGTATCCAATGTTAT | | | | |
| D1S4940-Rev-Primer Sequence: 5’GCGGAGGTTACGGTGAGCGAAGAT | | | | |
|  | | | | |
| D1S4946-Fwd-Primer Sequence: 5’6-FAM-AAGTAGCTGGGAATCCCTGT | | | | |
| D1S4946-Rev-Primer Sequence: 5’CCACCACTCCCGCCTAATTT | | | | |
|  | | | | |
| D1SPYGM-Fwd-Primer Sequence: 5’6-FAM-CTAGCCAGAGTCCACCTACTG | | | | |
| D1SPYGM-Rev-Primer Sequence: 5’GCTGTCAGGTAGCAACTGAC | | | | |

Supplementary Figure S1 Results of samples in which LOH status was not obtained are shown.

The results obtained for *PHLDA3* (A) and *MEN1* (B) gene locus are shown. LOH status was unavailable because of failure of PCR due to insufficiency, low quality of DNA (shown in green columns), microsatellite instability (shown in yellow columns), homozygosity (shown in gray columns), or unavailability of the samples (shown in pink columns).
